# Reliability and convergent validity of the PHQ-2 for the rapid detection of depressive symptoms in healthcare professionals in Argentina

**DOI:** 10.3389/fpsyt.2025.1652072

**Published:** 2025-11-28

**Authors:** Nicole Caldichoury, Breiner Morales-Asencio, Juan-Carlos Coronado, Luis Mario Castellanos-Alvarenga, César Quispe-Ayala, Carol Saldías, David Salazar, Daniela Ripoll-Córdoba, Wendy Bada, Juan Martínez, Rodrigo Duhalde-Sanhueza, Cesar Castellanos, Yuliana Flórez, Raúl Quincho-Apumayta, Carlos Ardila-Duarte, Alberto Rivelino Patiño-Rivera, Pascual A. Gargiulo, Juan Cárdenas, Norman López

**Affiliations:** 1 Departamento de Ciencias Sociales, Universidad de Los Lagos, Osorno, Chile; 2 Consorcio Latinoamericano de Investigación (CLATI), Temuco, Chile; 3 Departamento de Procesos Terapéuticos, Facultad de Ciencias de la Salud, Universidad Católica de Temuco, Temuco, Chile; 4 Escuela de Psicología, Facultad de Ciencias Sociales y Comunicaciones, Universidad Santo Tomás, Temuco, Chile; 5 Universidad Nacional de Huancavelica, Huancavelica, Peru; 6 Escuela de Kinesiología, Facultad de Salud, Universidad Santo Tomas, Temuco, Chile; 7 Universidad Nacional Daniel Alcides Carrión, Cerro de Pasco, Peru; 8 Universidad Nacional Intercultural de la Amazonia, Pucallpa, Peru; 9 Huertas College, Caguas, Puerto Rico; 10 Coordinador de campos Clínicos, Universidad Santo Tomás, Temuco, Chile; 11 Instituto Dominicano para el Estudio de la Salud Integral y la Psicología Aplicada (IDESIP), Santo Domingo, Dominican Republic; 12 Departamento de Ciencias Sociales, Universidad de La Costa, Barranquilla, Colombia; 13 Departamento de Ciencias Básicas, Universidad Metropolitana, Barranquilla, Colombia; 14 Facultad de Educación, Universidad Nacional Intercultural de la Selva Central Juan Santos Atahualpa, La Merced, Chanchamayo, Peru; 15 Laboratorio de Neurociencias y Psicología Experimental, Área de Farmacología, Facultad de Ciencias Médicas, Universidad Nacional de Cuyo, Mendoza, Argentina; 16 Universidad Nacional Autónoma Altoandina de Tarma,Tarma, Peru

**Keywords:** depression, healthcare professionals, validation, test, PHQ-2 screening

## Abstract

**Introduction:**

The prevalence of depressive symptoms among healthcare professionals has increased significantly, highlighting the need for valid and reliable ultra-rapid screening tools in high-demand clinical settings.

**Objective:**

To analyze the reliability and convergent validity of the Patient Health Questionnaire-2 (PHQ-2) among healthcare professionals in Argentina.

**Method:**

A cross-sectional eHealth study was conducted with 2,835 healthcare professionals (47% men, 53% women) working in public (57.2%) and private (42.8%) institutions. Participants completed the PHQ-2, PHQ-9, GAD-7, and Mini-Z scales in two phases, with a three-month interval. The convergent validity of the test was determined by comparing it with the original version of the PHQ, an anxiety test (GAD-7), and a burnout test (Mini-Z). In addition, internal consistency was calculated using Cronbach's alpha coefficient and McDonald's omega coefficient.

**Results:**

The PHQ-2 showed a unidimensional structure, explaining 73% of the variance, and demonstrated strong convergent validity, with high correlations with the PHQ-9 (r = 0.836; p < 0.001) and the GAD-7 (r = 0.724; p < 0.001), and a moderate correlation with the Mini-Z (r = 0.568; p < 0.001). Internal consistency was satisfactory (α = 0.75; ω = 0.85), confirming its reliability as a screening tool.

**Conclusions:**

The PHQ-2 is a valid and reliable instrument for the ultra-rapid detection of depressive symptoms in healthcare professionals in Argentina. Its strong psychometric properties and brief format make it a useful tool for mental health screening in high-demand healthcare settings.

## 1 Introduction

The COVID-19 pandemic caused a widespread deterioration of mental health worldwide; anxiety and depression became one of the most recurrent emotional disorders (1–3). In particular, healthcare professionals faced exceptional psychological demands throughout the pandemic, resulting from continuous clinical duties, increased workloads, and the absence of clear expectations about how the crisis would evolve (4–6). In this context, emotional disorders such as depression became some of the most disabling mental conditions, affecting both individual well-being and professional performance (7–9).

In the general population, the prevalence of depressive symptoms reached 28.18% during the pandemic and decreased to 20.0% in the post-pandemic period (10, 11). These rates were consistently higher among healthcare professionals, who exhibited greater levels of depressive symptoms in both phases.

Recent studies have reported that between 22.8% and 37.1% of healthcare professionals experienced depressive symptoms during the pandemic (12, 13). In the post-pandemic period, the figures ranged from 20.5% to 50.7%, consistently exceeding the rates observed in the general population (14–16). However, in Latin America the situation was even more critical, due to the structural fragility of healthcare systems, the scarcity of resources, and political instability; factors that exacerbated the psychological burden on healthcare workers (17, 18). In countries such as Argentina, Colombia, and Peru, the prevalence of depressive symptoms among healthcare personnel ranged between 59.18% and 91.7% (19–21); with an increase in the incidence of occupational burnout (59.8%) and cognitive dysfunctions (69.2%), associated with the care of patients infected with coronavirus (22, 23).

The repercussions of depression in this group go beyond the individual level. Several studies have shown that depressive symptoms are linked to concentration difficulties, which increase the risk of medical errors and reduce the quality of care (23, 24). These results emphasize the need for rapid and effective tools to detect depressive symptoms among healthcare professionals. This is particularly relevant in hospital environments, where high clinical workloads and limited resources often hinder the implementation of systematic assessments (25–27).

The PHQ-2 is the abbreviated version of the Patient Health Questionnaire-9 (PHQ-9). It consists of two items that evaluate the core symptoms of depression, depressed mood and anhedonia, as recognized by the DSM (28, 29). This brief version demonstrated adequate reliability and convergent validity across different clinical contexts. In the original validation study, conducted in eight primary care clinics and seven obstetrics and gynecology centers, it achieved a sensitivity of 83% and a specificity of 90% for major depression (28). Subsequently, similar diagnostic accuracy was confirmed, with a sensitivity of 86% and a specificity of 78% in comparable clinical samples (30). Likewise, a study reported an area under the curve (AUC) of 0.84, and a meta-analysis including more than 40,000 participants estimated an overall AUC of 0.89 (31, 32). In Latin America, studies in rural Mexico and Brazil also confirmed its validity. The Mexican study reported a sensitivity of 80% and a specificity of 87%, whereas the Brazilian sample of women in primary care demonstrated adequate discriminant validity (33, 34).

Moreover, recent research in hospital and community settings has confirmed the usefulness of the PHQ-2 as a brief screening tool, emphasizing its value in contexts with limited time and resources (35–37). However, in Argentina, there are still no reports describing its psychometric properties. Across Latin America, the only formal validation published to date refers to healthcare professionals in Colombia (38). That investigation, conducted with 725 physicians and nurses during the COVID-19 pandemic, demonstrated high convergent validity between the PHQ-2 and PHQ-9 (r = 0.860; p < 0.001), adequate internal consistency (α = 0.80; ω = 0.76), and strong item–total correlations (r = 0.910 and r = 0.924). These results confirm that the PHQ-2 is a reliable and valid instrument for the rapid detection of depressive symptoms in high-demand healthcare settings. Therefore, this research aimed to analyze its reliability and convergent validity among Argentine healthcare professionals.

## 2 Materials and methods

This study constitutes a secondary analysis of original data obtained from an international multicenter project that evaluated mental health indicators in Latin America and the Caribbean following the COVID-19 pandemic (INV.140-03-001-18). Between May 12, 2022, and November 27, 2023, data were collected through an electronic questionnaire administered in two stages. The instrument was distributed using a snowball sampling strategy. The overall sample included more than 23,000 participants from 15 Latin American countries, encompassing healthcare professionals, outpatients, university students, and community members from diverse occupational backgrounds.

This analysis examined the internal reliability of the PHQ-2 and its construct validity through factor analysis. Convergent validity was also tested using correlations with the PHQ-9, GAD-7, and Mini-Z scales, allowing for the assessment of its internal structure and its relationship with depression, anxiety, and burnout.

### 2.1 Participants

From the total database, 3,931 records corresponded to physicians and nurses from Argentina. Among them, 2,948 participants (75%) were selected because they had completed the initial form, and their identity and professional role were verified through cross-validation of personal and institutional email addresses. However, only 2,835 subjects completed the second digital form, which therefore constituted the final analyzed sample. The sample size included all valid available cases, with no *a priori* estimation.

### 2.2 Procedure

In the original study, an automated Google form was used that included a unique identifier for each subject, demographic data (age, sex, type of profession, employment status, and institutional email). Additionally, questions on lifestyle habits and health conditions were included, along with the administration of two psychometric instruments: the PHQ-9 and the GAD-7. To date, two publications have documented the psychometric properties of one of the instruments used in this research (2, 39).

From the 3,931 initial records of Argentine healthcare personnel, 75% (n = 2,948) were randomly selected through an automated algorithm that prioritized verifiable personal and institutional email addresses. This subsample was used to cross-validate identity and data consistency according to methodological criteria, which optimized internal validity and reduced potential bias. Operational criteria were also applied to ensure a manageable response rate and an appropriate logistical workload. Finally, 2,835 subjects completed the second digital form, constituting the final analyzed sample.

The selected participants received a second questionnaire that included new questions on lifestyle and health conditions, along with the Mini-Z and PHQ-2 instruments. The estimated duration of the first assessment was approximately eight minutes, while the second required about six minutes. A minimum interval of three months was established between both phases based on two main considerations. First, to reduce the possibility of learning or recall bias resulting from the repetition of similar items between the PHQ-9 and PHQ-2. Second, to accommodate the logistical conditions of the multicenter project (INV.140-03-001-18), which was implemented in successive stages during the pandemic and, in several cases, made the simultaneous administration of both instruments unfeasible.

To minimize selection bias, follow-up communications were sent only to participants who had completed the first phase and provided valid email addresses, allowing personal and institutional cross-validation. This measure reduced the risk of identification errors and contributed to maintaining the integrity of the longitudinal data. Additionally, to prevent multiple submissions, the Google Form was configured to accept only one response per institutional email account. This restriction was complemented by a manual review of duplicates through the comparison of unique identifiers and key demographic variables, further reinforcing the reliability and uniqueness of the data collection process.

In the first assessment phase, 983 records were excluded (3,931 → 2,948), and in the second phase, an additional 113 participants (2,948 → 2,835) were removed. These exclusions were due to unverified professional identity, incomplete forms, duplicate records, or multiple entries with inconsistent identifiers.

### 2.3 Instrument

The PHQ-2 is a brief tool derived from the PHQ-9 (28). It consists of two items that evaluate depressed mood and anhedonia over the previous two weeks. Each item is rated on a scale from 0 ("not at all") to 3 ("nearly every day"), with a total score ranging from 0 to 6. These two items represent the core symptoms of depression defined by the DSM diagnostic criteria and form the diagnostic basis of a major depressive episode. The original proposal has been replicated in multiple settings, demonstrating that this ultra-brief version maintains adequate validity and clinical utility (28). The validation of the Spanish version in a Colombian population, specifically among healthcare professionals, reported high internal consistency (Cronbach’s α = 0.80) (38). A cutoff score of ≥ 3 has been recommended as an indicator of major depression, supported by robust meta-analytic evidence (40).

To assess the convergent validity of PHQ-2, three measures associated with depressive symptoms were used. First, the original nine-item version of PHQ was administered (41). This scale measures the frequency of depressive symptoms over the previous two weeks. Each item is scored from 0 (“not at all”) to 3 (“nearly every day”), yielding a total score from 0 to 27. Scores of 10 or higher indicate moderate to severe depression. The PHQ-9 has demonstrated excellent psychometric properties and has been validated in a wide range of clinical and population-based studies worldwide (42–44).

Likewise, the Generalized Anxiety Disorder scale (GAD-7) was used (45). It is a brief seven-item instrument that assesses symptoms of generalized anxiety over the previous two weeks. Each item is rated on a Likert scale from 0 (“not at all”) to 3 (“nearly every day”), yielding a total score ranging from 0 to 21. Scores of 10 or higher indicate moderate to severe anxiety. The Spanish version has shown adequate validity among healthcare professionals in Colombia and Bolivia (46). It is a widely used measure in Latin America and the Caribbean, with excellent psychometric properties (2, 39).

Finally, the Mini-Z scale was administered (47, 48). This brief 10-item instrument assesses burnout and psychosocial factors in the workplace, including job satisfaction, workload, perceived control, stress, and alignment with institutional values. Item 1 evaluates emotional exhaustion and is considered the main indicator of burnout, with scores ≥ 3 indicating clinical risk. Responses are recorded using Likert or dichotomous formats, as appropriate. The Spanish version for Spanish-speaking populations, developed by our working research team, was used (49).

The inclusion of the GAD-7 and the Mini-Z aimed to examine the convergent validity of PHQ-2 with emotional constructs closely related to depression, such as anxiety and burnout. Several studies have demonstrated that these conditions often coexist among healthcare professionals, although they represent distinct constructs (50–52). This approach allowed us to analyze whether PHQ-2 was significantly associated with related emotional states, reinforcing its convergent validity without compromising its specificity as a depression screening tool.

### 2.4 Data analysis

A multi-stage statistical analysis was conducted to assess the psychometric properties of the PHQ-2. First, descriptive statistics were calculated for each item, including mean (M), standard deviation (SD), kurtosis, skewness, and 95% confidence intervals. Subsequently, convergent validity was examined using Pearson’s correlation coefficients between the total PHQ-2 score and the PHQ-9, GAD-7, and Mini-Z scores. This procedure aimed to verify whether the PHQ-2 measured the same theoretical construct related to depressive symptomatology. Although the items followed a Likert-type format, the total score was treated as a continuous variable, in accordance with widely accepted psychometric standards (53).

The normality of score distributions was assessed using the Kolmogorov–Smirnov test, which was nonsignificant and therefore supported the use of parametric statistics. Consequently, Pearson’s correlations were performed, and the use of Spearman’s coefficients was ruled out since no assumption violations were detected. Internal consistency was then estimated through Cronbach’s alpha and McDonald’s omega coefficients, as well as corrected item–total correlations, to determine item homogeneity.

Additionally, the unidimensionality of the PHQ-2 was explored through a factor analysis, acknowledging that with two items this procedure yields a just-identified model (with no degrees of freedom). Therefore, the analysis was interpreted solely as complementary evidence (focused on factor loadings and inter-item correlation), and internal validity was primarily supported by reliability and convergent validity, as recommended by scale development guidelines (54, 55). According to structural modeling guidelines, two-indicator factors may be used for confirmatory purposes under these precautions and with limited interpretation (56, 57). All analyses were conducted using R software, version 1.3.1056.

### 2.5 Ethical considerations

Participants were previously informed about the objectives of the study and provided their informed consent through an online form. To ensure the accuracy of the information, each individual’s participation and professional role were verified via telephone and email contact. Additionally, participants were provided with a document containing psychological recommendations for managing stress and depressive symptoms. This consent also included the possibility that their data could be used in subsequent analyses for research purposes, including the validation of psychometric instruments. The study was conducted in accordance with national and international ethical guidelines for research involving human beings, in line with the Declaration of Helsinki of 1975, revised in 2008. The project was approved by the ethics committee of Universidad de La Costa (Code 086/2020 and 173/2022) which led the study.

## 3 Results

Participants ranged in age from 29.18 to 47.04 years (M = 39.22, SD = 13.61); 47% were men (n = 1,332) and 53% were women (n = 1,503). Regarding professional category, 28.36% were general practitioners, 37.24% specialists, and 34.4% nursing staff. Among the total participants, 42.8% worked in private institutions and 57.2% in the public healthcare system. **Table 1** shows that the two PHQ-2 items presented similar means, indicating consistency in responses. Likewise, the distributions were approximately symmetric, without pronounced tails, and the narrow confidence intervals demonstrate the stability of the estimates.

**Table 1:** Descriptives of the PHQ-2.

Items	ME	DE	Kurtosis	Skewness	Confidence interval	
					Lower limit	Upper limit
Depressed mood	1.11	0.85	-0.25	0.50	1.05	1.17
Anhedonia	1.07	0.88	-0.19	0.63	1.00	1.13

ME, Mean, SD, Standard Deviation

The confirmatory factor analysis (CFA) results (**Table 2**) supported the unidimensional structure of the PHQ-2. Because this model is just-identified (with no degrees of freedom), global fit indices (χ², RMSEA, CFI, SRMR) were not reported, as they are uninformative for this type of structure. Instead, emphasis was placed on the factor loadings and inter-item correlation as evidence of unidimensionality.

**Table 2:** Confirmatory factor analysis.

Item	Standard loading (λ)	Error (ϵ)	Explained variance (λ²
Depressed mood	0.82	0.29	0.71
Anhedonia	0.84	0.28	0.73

The Confirmatory Factor Analysis (CFA) model of the PHQ-2 showed high standardized loadings for both items (λ = 0.82 and λ = 0.84), indicating strong saturation on the latent depression factor. The associated errors (ϵ = 0.29 and 0.28) were low, suggesting little unexplained residual variance. Similarly, the variance explained by each item was high (λ² = 0.71 and 0.73, respectively), supporting the stability of the proposed model. These results were interpreted as complementary evidence of unidimensionality, while construct validity was primarily supported by internal consistency and convergent validity.

### 3.1 Convergent validity

Then, correlations were conducted between the PHQ-2 and three relevant measures: the original version of the PHQ-9, the Generalized Anxiety Disorder test (GAD-7), and a Burnout Test (Mini-Z) (see **Table 3**). A very strong correlation was observed with the PHQ-9 (r = 0.836; p < 0.001), confirming that both instruments assess the same theoretical construct. Likewise, a high correlation was identified with the GAD-7 (r = 0.724; p < 0.001) and a moderate but significant correlation with the Mini-Z (r = 0.568; p < 0.001). This differential pattern of magnitudes is consistent with the convergent validity of the PHQ-2 and indicates a stronger alignment with depressive symptomatology than with anxiety or burnout; however, given shared internalizing variance and the two-item format, these findings should be interpreted as convergence predominance rather than strict discriminant validity or diagnostic specificity.

**Table 3:** Correlation of PHQ-2 with mini-Z, GAD-7, and PHQ-9 measures.

	PHQ 9	GAD-7	Mini-Z
PHQ 2	,836^**^	,724**	,568**

**p<0.01

### 3.2 Reliability analysis

Subsequently, internal consistency analyses of the PHQ-2 were performed. As shown in **Table 4**, the instrument demonstrated acceptable reliability, with a Cronbach’s alpha coefficient of 0.75 (95% CI: 0.70–0.79), indicating adequate response consistency. The item-total correlations were high, with values of 0.889 for Item 1 and 0.898 for Item 2, both significantly above the recommended minimum threshold of 0.30. These results reflect a homogeneous internal structure, supporting the stability and reliability of the PHQ-2 for use in this population.

**Table 4:** Descriptive statistics and internal consistency of the PHQ-2.

	ME	DE	Item-total correlation
Depressed mood	1.11	0.85	0.889**
Anhedonia	1.07	0.88	0.898**

Cronbach’s Alpha = 0.75 (95% CI: 0.72 – 0.78); McDonald’s Omega = 0.77. M, Mean, SD, Standard deviation. ** indicates that the correlation is statistically significant at p < 0.01.

## 4 Discussion

We evaluated the reliability and convergent validity of the PHQ-2 for the rapid detection of depressive symptoms among Argentine healthcare professionals. The results showed a unidimensional structure, suggesting that the two test items assess a single construct. The corrected item–total correlations (0.889 and 0.898) substantially exceeded the recommended threshold, showing that both items contribute similarly and robustly to the measurement of depression. Additionally, the descriptive indicators showed approximate symmetry (skewness near zero) and low kurtosis, suggesting the absence of bias in item interpretation and providing additional evidence of the instrument’s stability in application. Nevertheless, because the PHQ-2 is a two-item scale, its CFA results should be interpreted with caution; models of such low complexity provide limited information, and beyond the inter-item correlation they do not allow a meaningful assessment of global fit (58). Therefore, in our study the CFA was used as complementary evidence of unidimensionality, whereas construct validity was primarily supported by reliability and convergent validity analyses (54, 55). A previous study warned that the interpretation of fit indices in simple models depends on sample size, number of items, and structural complexity (59). Even so, a recent validation of the PHQ-4 in a Chilean population has shown that, under conditions of high inter-item correlation and large sample sizes, two-indicator factors can be psychometrically acceptable (60).

Beyond methodological limitations, the psychometric robustness of the PHQ-2 is supported both theoretically and clinically. Its two items, focused on the core symptoms of depression (depressed mood and anhedonia), provide an efficient and accurate assessment of depressive symptomatology, to the extent that it is considered a reliable and valid ultra-brief instrument for detecting major depression (31, 61, 62). Despite its brevity, it achieves high levels of sensitivity (S) and specificity (E), and its clinical utility (AUC) has been widely demonstrated in both clinical and community settings (63–67). In this regard, an individual participant data meta-analysis with over 40,000 participants estimated an overall AUC close to 0.90, with high levels of S and E compared to diagnostic interviews (31). Complementarily, a recent meta-analysis in Spanish-speaking populations confirmed equivalent performance, with pooled S and E indicators of 0.89 and an AUC of 0.87, reinforcing its clinical validity in Latin America and Spanish-speaking communities (68).

Regional studies have shown that the PHQ-2 is a valid and useful instrument for the rapid detection of depressive symptoms. In Chile, found that a cut-off score of ≥3 yielded a sensitivity of 74.6%, a specificity of 93.9%, and an AUC of 0.92 in a rural population (69). Among patients with chronic illnesses, another study reported a diagnostic accuracy of 0.92 for detecting depression cases (67). In Mexico, a community-based study observed comparable performance, with a sensitivity of 80.0%, specificity of 86.9%, and an AUC of 0.89 using a cut-off score of 3 (33). Finally, in Spain, confirmed its validity in primary care, establishing an optimal cut-off score of ≥2 and corroborating its unidimensional structure in the general population (70). This evidence reinforces the clinical utility of the PHQ-2 as a brief, reliable, and valid instrument for the rapid detection of depressive symptoms across various Spanish-speaking contexts, both within and beyond hospital settings.

On the other hand, the convergence of the PHQ-2 with other instruments capturing emotional dimensions related to depression in hospital settings was examined-specifically the PHQ-9, GAD-7, and Mini-Z. First, a high correlation was observed with the PHQ-9 (r = 0.836; p < 0.001), confirming that the two core items of the PHQ-2 accurately reflect the depressive construct assessed by the full scale. This result reinforces its convergent validity and aligns with international evidence, which has documented consistent associations across various populations and clinical settings.

In Chinese populations, very high correlations have been reported between the PHQ-2 and the PHQ-9, both among adolescents (r = 0.86–0.89) and among older adults living in rural areas (71). In primary care settings, a study conducted in Ethiopia reported a strong correlation (r = 0.83), while research from Saudi Arabia also found a high correlation (r = 0.79; R² = 0.62) (72). These findings highlight the ability of the PHQ-2 to function as a brief and effective screening tool in everyday clinical practice settings.

Additionally, adequate levels of convergence between the PHQ-2 and the PHQ-9 have been documented in specific clinical populations, such as patients with heart failure (73), migraine (74), adults with chronic obstructive pulmonary disease (75), and women with infertility (64).

In the Latin American context, a study conducted in Costa Rica reported a very high correlation (r = 0.843; p < 0.001) between the PHQ-9 and GAD-7 in the general population (76); and another conducted in Colombia identified a significant association (ρ = 0.70; p < 0.01) between PHQ-9 and PHQ-2 among primary care users (77). Finally, the only research to date in a population of healthcare professionals in Latin America that has analyzed the correlation between the PHQ-2 and the PHQ-9 was conducted in Colombia (38). This research reported a high correlation (r = 0.860; p < 0.001), reinforcing the robustness of the PHQ-2 as a brief and valid instrument for screening depressive symptoms in high-demand clinical environments such as hospitals and workplaces.

Secondly, the PHQ-2 demonstrated adequate convergence with the GAD-7 (r = 0.724; p < 0.001), indicating that it also captures emotional distress dimensions related to anxiety. This finding is consistent with evidence reported across different international contexts. In Chile, a similar association was observed among migrants (65). In Germany, a moderate correlation (r = 0.64) was reported in both the general population and primary care, confirming the frequent comorbidity between brief depression and generalized anxiety (78). In Iran, significant correlations were identified in patients with infertility, supporting the usefulness of the PHQ-2 in clinical contexts linked to anxiety (79). Consistent correlations between the PHQ-2 and GAD-7 have also been reported among U.S. college students, ranging from ρ = 0.45 to 0.65, supporting the stability of this relationship in younger populations (80).

Thirdly, the PHQ-2 showed a moderate correlation with the Mini-Z (r = 0.568; p < 0.001), supporting its applicability in identifying emotional distress associated with work overload and occupational stress. In Puerto Rico, a study of employees found a positive association between depression and the emotional exhaustion and cynicism dimensions of the Maslach Burnout Inventory (81). Furthermore, moderate correlations among burnout, depression, and anxiety have been reported, confirming that these are related yet conceptually distinct constructs (50).

In hospital settings, this link is reinforced by the role of chronic stress due to work overload in the development of emotional problems such as anxiety and, ultimately, depression. Evidence indicates that prolonged exposure to stress activates the hypothalamic–pituitary–adrenal (HPA) axis, increasing cortisol release and altering neuronal plasticity in the hippocampus and prefrontal cortex (82). These alterations impair emotional regulation and executive functioning, leading to poorer decision-making and the emergence of depressive symptoms (83). Moreover, burnout has been associated with reduced levels of Brain-Derived Neurotrophic Factor (BDNF), a neurotrophin essential for resilience, whose decrease exacerbates cognitive and emotional dysfunction (84). The convergence of these mechanisms reinforces the relationship between chronic occupational stress and vulnerability to depression (85).

Taken together, the available evidence and our findings confirm that the PHQ-2 captures a depressive core closely linked to burnout and other mechanisms related to occupational stress, reinforcing its psychometric robustness and clinical applicability in high-demand healthcare settings.

Finally, the PHQ-2 demonstrated adequate reliability, with a Cronbach’s alpha coefficient of 0.75 and a McDonald’s omega coefficient of 0.85, indicating satisfactory internal consistency. These results are consistent with those obtained in previous validations conducted among healthcare professionals. In Colombia, a study reported an alpha of 0.80 and an omega of 0.76 among medical and nursing staff (38). In Chile, similar coefficients of 0.78 and 0.79 were observed in a rural community sample (69). The similarity of these findings reinforces the psychometric stability of the PHQ-2 as a reliable tool.

Undoubtedly, the PHQ-2 could be successfully integrated among ultra-rapid depression screening tests, as it maintains its diagnostic validity even in its abbreviated two-item version (36, 86). Indeed, the simplification of assessment scales is a key strategy for optimizing the detection of depressive symptoms without compromising validity (87). Its use in time-constrained settings, common in some hospital services, reduces the burden on both professionals and patients, ensuring accurate detection. In this context, the PHQ-2 facilitates the early identification of at-risk cases and supports associated interventions.

This trend toward brief assessment tools is also evident in instruments such as the Beck Depression Inventory–FastScreen (BDI-FS) and the 10-item Center for Epidemiologic Studies Depression Scale (CES-D-10), abbreviated versions of the BDI-II and CES-D. Both have shown strong psychometric performance, maintaining sensitivity and specificity levels comparable to their original versions (88–90). Their usefulness has been demonstrated in occupational environments characterized by high exposure to stressors, which underscores the relevance of our findings. In this regard, the PHQ-2 can be included among ultra-brief tools with clinical validity for detecting depressive symptoms across diverse contexts, distinguished by its simplicity and ease of use.

However, our study presents some limitations that should be noted. First, the cross-sectional design prevents the establishment of causal relationships between the variables analyzed; moreover, the premorbid status of the participants was not known. The sampling, although appropriate for this type of population, may limit the generalizability of the findings to all healthcare professionals in Argentina. Additionally, factorial invariance by sex was not assessed, which could be explored in future research to strengthen the structural validity of the instrument.

It should also be acknowledged that the factor analysis applied to the PHQ-2 has a limited interpretative scope, since two-item models are just-identified and lack degrees of freedom. Therefore, this procedure was used solely as complementary evidence of unidimensionality; whereas internal validity relied mainly on internal consistency and convergent validity indices.

Another limitation concerns the three-month interval between the PHQ-9 and PHQ-2 administrations. Although this separation helped minimize potential memory effects from repeated items and was consistent with the multicenter study’s data collection schedule, it may also have led to variations in participants’ depressive symptoms. Therefore, convergent validity results should be interpreted with caution, and future studies should apply both versions concurrently to obtain more accurate estimates.

Similarly, a formal analysis of divergent validity was not conducted, which limits the ability to confirm that the PHQ-2 specifically measures depressive symptoms and not overlapping constructs such as anxiety or burnout. Future research should incorporate measures of divergent validity to strengthen the psychometric evidence of the instrument.

Finally, online administration may have limited the participation of professionals with restricted access to digital resources, introducing a potential selection bias. Future studies could adopt mixed methodologies, combining online surveys with in-person data collection to improve sample representativeness. Likewise, it would be relevant to compare the PHQ-2 with other ultra-rapid scales to assess its diagnostic accuracy across different clinical contexts.

Despite these limitations, the present study provides consistent psychometric evidence supporting the reliability and validity of the PHQ-2 among healthcare professionals in Argentina. Its strong internal consistency and high correlation with the original nine-item version (PHQ-9) support its convergent validity, confirming that both instruments assess the same depressive construct. In addition, the moderate associations observed with anxiety and burnout indicators reinforce its concurrent validity, demonstrating that the PHQ-2 can also capture emotional dimensions frequently intertwined with depressive symptomatology. Together, these findings confirm the theoretical coherence and measurement stability of the instrument. Although future studies should continue to examine its factorial invariance and temporal stability, the PHQ-2 stands out as a brief and reliable measure that could be useful for the rapid detection of depressive symptoms in healthcare populations.

## Data Availability

The raw data supporting the conclusions of this article will be made available by the authors, without undue reservation.
